# Protective Effect of *Bifidobacterium lactis* JYBR-190 on Intestinal Mucosal Damage in Chicks Infected With *Salmonella pullorum*

**DOI:** 10.3389/fvets.2022.879805

**Published:** 2022-05-27

**Authors:** Liangyu Yang, Yuanhong Chen, Qian Bai, Xi Chen, Yunteng Shao, Ronghai Wang, Fengping He, Ganzhen Deng

**Affiliations:** ^1^Department of Clinical Veterinary Medicine, College of Veterinary Medicine, Huazhong Agricultural University, Wuhan, China; ^2^Department of Clinical Veterinary Medicine, College of Veterinary Medicine, Yunnan Agricultural University, Kunming, China

**Keywords:** *Salmonella pullorum*, *Bifidobacterium lactis*, intestinal mucosa, inflammation, damage

## Abstract

Pullorum is one of the most serious diseases that endanger the chicken industry. With the advent of the era of anti-antibiotics in feed, the replacement of antibiotics by probiotics has become the focus and hotspot of related research. In this study, hematoxylin-eosin (H&E) staining, immunohistochemistry (IHC) and enzyme-linked immunosorbent assay (ELISA) were used to observe the structural changes of intestinal mucosa in chicks infected with *Salmonella pullorum*, and to analyze TNF-α, IL-10, IFN-γ, proliferating cell nuclear antigen (PCNA), and secreted immunoglobulin A (sIgA) levels. The results showed that the intestinal villus height, villus height to crypt depth ratio (V/C), and muscle layer thickness of duodenum, jejunum and cecum in the JYBR-190 group were significantly higher than those of the infection group and antibiotic group. Furthermore, the levels of PCNA, sIgA and IL-10 in JYBR-190 group were significantly increased, whereas the expression of TNF-α and IFN-γ was significantly decreased. Taken together, *Bifidobacterium lactis* JYBR-190 has a protective effect on intestinal mucosal damage in chicks infected with *Salmonella pullorum*.

## Introduction

Pullorum is a widespread acute intestinal infectious disease caused by Salmonella Pullorum in the world. Salmonella pullorum was discovered by Rettger (1), and it can be transmitted vertically to the next generation of chicks. The morbidity and mortality of 2–3 week old chicks are extremely high (2), causing serious impact on the world chicken industry (3–5). In recent years, many countries have restricted the use of antibiotics to varying degrees due to the overuse of antibiotics resulting in drug resistance of animal-derived bacteria and drug residues, and the “banning of antibiotics” in feed has become an inevitable trend in the development of international animal husbandry (6, 7). Therefore, probiotics have gradually entered people's field of vision as an alternative to antibiotics.

Probiotics improve food safety and animal gut health by producing organic acids, activating the host immune system, and producing antimicrobial agents (8). Studies have shown that probiotics play an important role in stabilizing intestinal flora and controlling pathogen proliferation, and the addition of probiotics to feed increases host and colonization resistance to enteric pathogens such as Salmonella and Campylobacter (9). Currently, Bifidobacterium strains such as Bifidobacterium longum, Bifidobacterium longum, Bifidobacterium longum, and Bifidobacterium suis have been used to modulate human and animal microflora (10, 11). Roselli et al. found that the mixture of Lactobacillus acidophilus and Bifidobacterium longum had a good preventive effect on trinitrobenzenesulfonic acid-induced colitis in mice. Zuo et al. found that Bifidobacterium infantis effectively attenuates colitis in mice by regulating Th1/Th17 cell-mediated immune responses (12–14). Other studies have shown that Bifidobacterium breve JCM1192 reduces the colonization of Salmonella typhimurium in the cecum by a competitive exclusion mechanism, and Bifidobacterium infantis BL2416 protects the gut by promoting the production of IFN-γ and TNF-α (15–17). Therefore, this study selected Bifidobacterium as the bacteria of interest to explore the protective effect of Bifidobacterium lactis JYBR-190 on the intestinal mucosa of chicks infected with S. pullorum.

## Materials and Methods

### Bacterial Strains

Salmonella pullorum was isolated and preserved in this experiment and fed with 2.32 × 10^9^ CFU at the median lethal dose (LD_50_) to induce pullorum infection. Bifidobacterium lactis JYBR-190 dry powder was donated by Shandong Zhongke Jia Yiwan Bioengineering Co., Ltd. It was fed via drinking water at the ratio of 10 g JYBR-190 dissolved in 100 L water to obtain a concentration of ~1 × 10^11^ CFU/mL.

### Animal Experiments

SPF fertilized eggs were purchased from Jinan Seth Poultry Technology Co., Ltd.. The hatching and animal experiments were performed in the laboratory of Yunnan Agricultural University. A total of 100 SPF chicks were randomly divided into five groups (*n* = 20): Group A, Group B, Group C, Group D, and Group E. Group A was fed a sterilized diet as the control group. Groups B, C, D, and E were orally administered 0.5 mL *S. pullorum* containing 2.32 × 10^9^ CFU from the age of 7 days. Meanwhile, Group C received JYBR-190 with a concentration of 1.0 × 10^9^ CFU/mL in the drinking water every day from the age of 1 day. JYBR-190 was first added to the drinking water of Group D every day after 24 h infection, and 0.15 g/kg of neomycin sulfate was added to the drinking water of group E every day after 24 h of infection. Each experimental group was reared in isolation, and the chicks were allowed to eat sterile diet and drinking water freely during the experiment. The mortality of chickens in each group from 7 days to 14 days was recorded, and the body weight of chickens in each group was weighed at 14 days of age. This study was approved by the Ethics Committee of Yunnan Agricultural University. All experimental procedures adhered to the institutional criteria for the care and use of laboratory animals.

### Sample Collection

At the age of 14 days, 5 chicks were randomly selected from each group, and their duodenum, jejunum and cecum of about 2 cm were collected, and the intestinal contents were washed with PBS, fixed in 4% paraformaldehyde, and used for hematoxylin- Eosin (H&E) staining and immunohistochemistry (IHC). Another about 5 cm of duodenum, jejunum and cecum were taken, and the intestinal segment was washed with PBS and ground into tissue homogenate for the determination of secreted immunoglobulin A (sIgA) and intestinal cytokines.

### Histological Analysis

Routine dehydration, clearing, paraffin immersion, and paraffin embedding were performed on fixed tissues. Two sets of 4 μm thick serial sections were prepared, one for H&E staining and the other for IHC staining. First soak the paraffin section in xylene twice for 8 min each time, then immerse in gradient alcohol for dewaxing, stain with hematoxylin for 3–5 min, differentiate with 1% hydrochloric acid-ethanol solution, turn blue with 1% ammonia solution, rinse with distilled water Sections were dehydrated with gradient alcohol, stained with eosin for 5 min, routinely dehydrated with paraffin sections, cleared with xylene for 5 min, and sealed with neutral gum. Two sections of the duodenum, jejunum and cecum of each chick were selected for sectioning, and three longest intestinal villi and crypts were selected for each section for measurement, and three visual fields were randomly selected to measure the thickness of the muscle layer. About 30 crypt depth/villus height and muscle thickness values were obtained for each group, and the whole intestinal section was imaged under a 20 × photographic microscope, each villus height and its corresponding crypt depth were measured, and this Ratio and mean of 3 sets of data values. The height of the intestinal villi was measured vertically from the tip of the intestinal villi to the opening of the intestinal gland; the depth of the crypt, the thickness of the muscle layer and the villus height were measured vertically from the opening of the intestinal gland to the mucosal muscle layer.

### Immunohistochemical Analysis

Sections were routinely deparaffinized with distilled water, incubated with 3% H_2_O_2_ for 10 min at room temperature (RT) to inhibit endogenous peroxidase activity. Antigen activity in the sections was repaired with sodium citrate buffer for 10 min, and incubated with 3% BSA at RT in a humidified chamber closed for 30min. After removing the serum, the rabbit anti-proliferating cell nuclear antigen (PCNA) antibody (BS-2007R, 1:20; Beijing Biotechnology, China) was added, and incubated overnight at 4°C, before adding biotinylated goat anti-rabbit antibody (K009; KemeiBorui Technology, Beijing, China) and incubating at 25°C for 1 h. Horseradish peroxidase (HRP)-labeled streptavidin complex (SP9002; Beijing Zhongshan Jinqiao Biotechnology Co., Ltd., Beijing) was added and incubated at RT for 30 min. 3, 3'-Diaminobenzidine (DAB; Beijing Zhongshan Jinqiao Biotechnology Co., Ltd., Beijing) was added for 5–10 min, and the degree of staining was controlled by a microscope. Counterstained with hematoxylin for 3 min, dehydrated and transparentized for 5 min, dried and sealed with neutral glue. 5 slices were selected for each group, and 40 × fields of view were selected for each slice. The mean optical density (OD) of intestinal PCNA immunopositive substances was determined using proplus (IPP) software 6.0.

### Measurement of Intestinal SIgA and Cytokine Levels

Five groups of intestinal samples were ground into a homogenate in liquid nitrogen, and sIgA levels were measured by chicken sIgA ELISA kit (Shanghai Enzyme Link Biotechnology Co., Ltd., Shanghai, China). IL-10, TNF-α, and IFN-γ levels were measured according to IL-10, TNF-α and IFN-γ ELISA kits (Shanghai Enzyme Link Biotechnology Co., Ltd., Shanghai, China).

### Statistical Analysis

Statistical analysis was performed using SPSS 13.0 (SPSS Inc., Chicago, IL). If the data obey normal distribution, one-way ANOVA is used; Otherwise, the multiple rank sum test is used.Data organization and analysis were performed with Graphpad Prism 8.0.1, and data measurements were performed using Image Proplus (IPP) software version 6.0. All data were expressed as mean ± standard deviation (SD) or percentage. *P* < 0.05 was considered statistically significant.

## Results

### Mortality Rate and Average Body Weight of Different Treatment Groups

At 14 days of age, the mortality rate of blank control group A was 10%, and the mortality rate increased to 70% after infection with Salmonella pullorum. Compared with the infection group, feeding JYBR-190 before infection reduced the mortality rate to 33.3%, and feeding JYBR-190 and antibiotic treatment after infection reduced the mortality rate to 45% and 40%. The average weight of the blank control group was 71.25 g, while the average weight was reduced to 44.62 g by infection with Salmonella pullorum. Compared with the infection group, feeding JYBR-190 before infection increased the average body weight to 76.79 g, and feeding JYBR-190 and antibiotic treatment after infection increased the average body weight to 60.02 g and 58.35 g, respectively. The results show that feeding Bifidobacterium lactis JYBR-190 can reduce the mortality and weight loss caused by *Salmonella pullorum* infection, and feeding JYBR-190 before infection is more effective.

### Pathological Changes of Duodenum, Jejunum and Cecum in Different Treatment Groups

**Figure 1:**
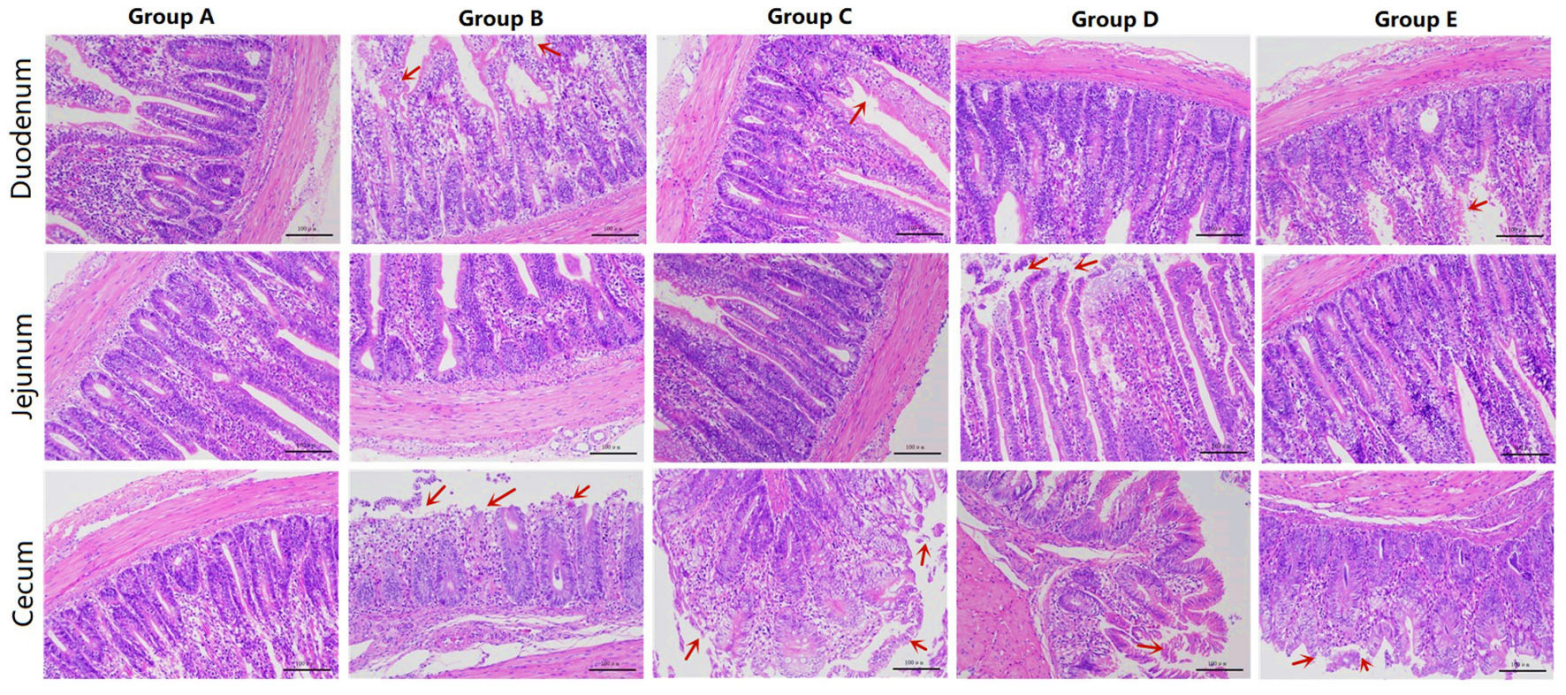
HE sections of different treatment groups. Group A, control group; Group B, infection group; Group C, JYBR-190 pre-fed group; Group D, JYBR-190 fed group; Group E, antibiotic group.

Morphological observation of the duodenum showed that the duodenal villi in the blank control group had a complete structure, neatly arranged epithelial cells, and no obvious pathological changes (Figure 1). In the infection group, the villi structure was incomplete, the villus epithelial cells were dissolved and disappeared, the arrangement was disordered, and the intestinal glands had mild hyperplasia. In the JYBR-190 pre-fed group, the villi structure was relatively complete, the epithelial cells in the upper segment of villus were slightly shed, and the intestinal glands had mild hyperplasia, but the degree of lesions was milder than that in the infection group. The JYBR-190-fed group had a complete villi structure, neatly arranged epithelial cells, no epithelial shedding, and infiltration of lamina propria inflammatory cells. In the antibiotic group, the villous structure was incomplete, the villous epithelium was shed, and the intestinal glands had mild hyperplasia.

The jejunum morphological structure showed that the jejunal villi in the blank control group had a complete structure, neatly arranged epithelial cells, and no obvious pathological changes such as epithelial shedding or inflammatory cell infiltration (Figure 1). In the infection group, the jejunal villus epithelial cells were sloughed off and the upper villus epithelial cells were sloughed off more obviously, the cells were not arranged neatly, some inflammatory cells infiltrated the lamina propria and the basal part, and the intestinal gland cells proliferated. In the JYBR-190 pre-fed group, the villi structure was complete, and the epithelial cells were slightly disordered. In the antibiotic group and the JYBR-190 fed group, the epithelium of the villi fell off, and there were no other obvious lesions.

The cecum morphological structure found that some epithelial cells of the cecum villi were shed in the blank control group (Figure 1), and the others had no obvious lesions. In the infection group, the structure of the cecal villi was incomplete, epithelial cells were severely shed, a large number of inflammatory cells infiltrated the lamina propria and basal layer, and intestinal gland cells proliferated. The villous structure of the JYBR-190 pre-fed group was relatively complete, and the epithelial cells in the upper segment of the villi were partially shed. The intestinal villi in the JYBR-190 fed group were damaged, and the villus epithelial cells were necrotic and shed. Villous epithelial cell shedding and intestinal glandular hyperplasia were observed in the antibiotic group.

### Changes in Villus Height, Crypt Depth, Villus Height and Crypt Depth, (V/C) Ratio and Muscle Thickness of Each Intestinal Segment in Different Treatment Groups

**Figure 2:**
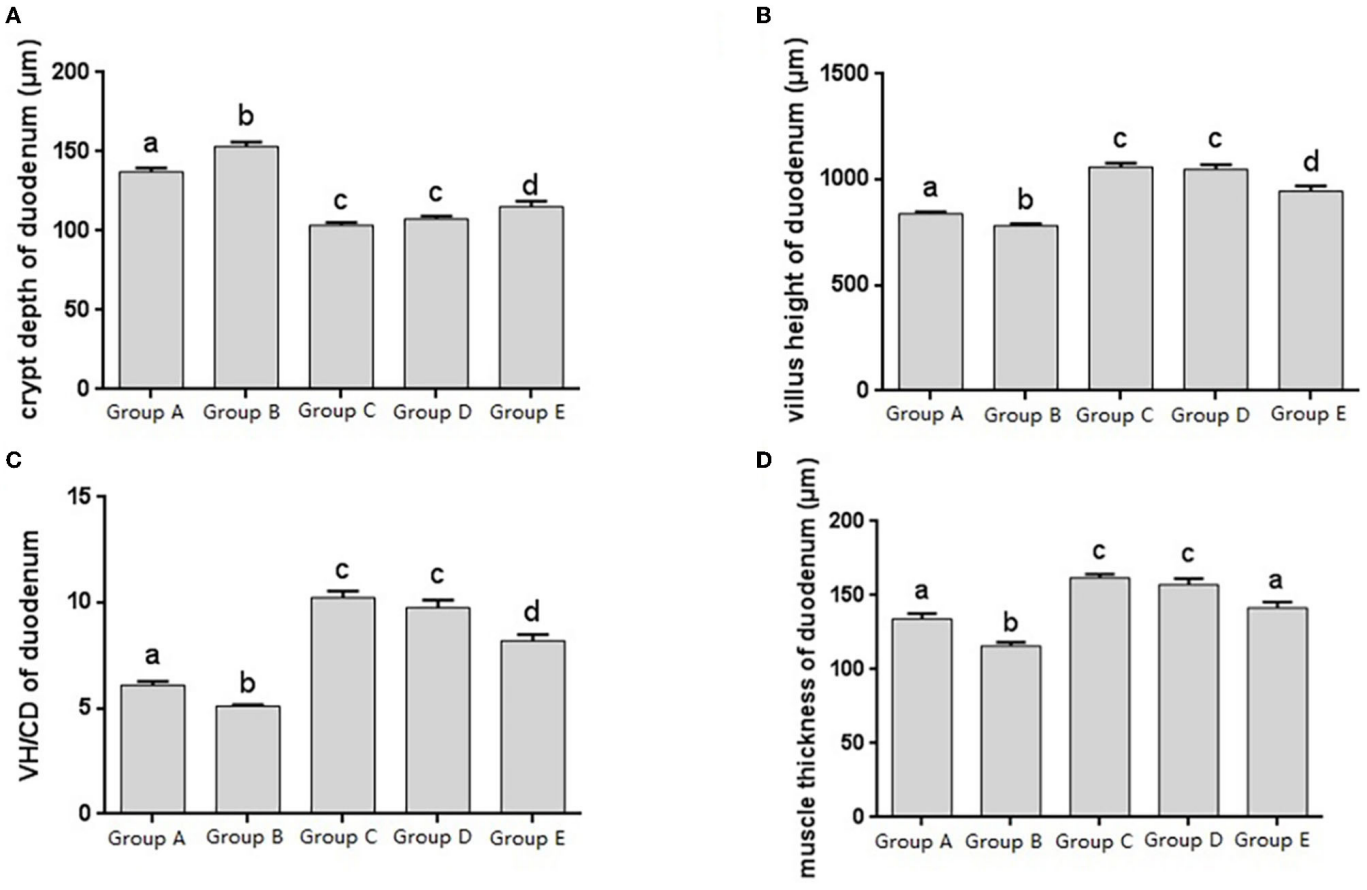
Morphological changes in the duodenum of chicks after different treatments. **(A)** Crypt depth (CD) of the duodenum. **(B)** Villus height (VH) of the duodenum. **(C)** VH/CD of the duodenum. **(D)** Muscle thickness of duodenum. Group A, control group; Group B, infection group; Group C, JYBR-190 pre-fed group; Group D, JYBR-190 fed group; Group E, antibiotic group. Data are expressed as mean ± standard deviation (SD). Different lowercase letters on the columns indicate significant differences (*P* < 0.05); the same lowercase letters indicate no significant difference (*P* > 0.05).

The histomorphology of duodenum in different treatment groups was observed, and the changes of duodenal villus height, crypt depth, V/C ratio and muscle layer thickness were analyzed. Changes in the depth of intestinal crypts showed that the depth of intestinal crypts in the Salmonella pullorum infection group was significantly higher than that in the blank control group. Compared with the Salmonella pullorum infection group, the depth of intestinal crypts in the JYBR-190 pre-fed, JYBR-190 fed, and antibiotic groups were reduced to varying degrees, and the difference was significant. The depth of the fossa decreased significantly (Figure 2A). Changes in intestinal villus height, V/C value and muscle layer thickness showed that the Salmonella pullorum infection decreased significantly the intestinal villus height, V/C value and muscle layer thickness compared with the blank control group. Compared with the Salmonella pullorum infection group, the intestinal villus height, V/C value and muscle layer thickness in the JYBR-190 pre-fed, JYBR-190 fed, and antibiotic groups were increased to different degrees, and the difference was significant. The intestinal villi, V/C value and muscle layer thickness were significantly higher in the JYBR-190 pre-fed and fed groups than in the antibiotic group (Figures 2B–D). It indicated that feeding Bifidobacterium lactis JYBR-190 could increase the duodenal villus height, V/C value and muscle layer thickness, and decrease the depth of intestinal crypts.

**Figure 3:**
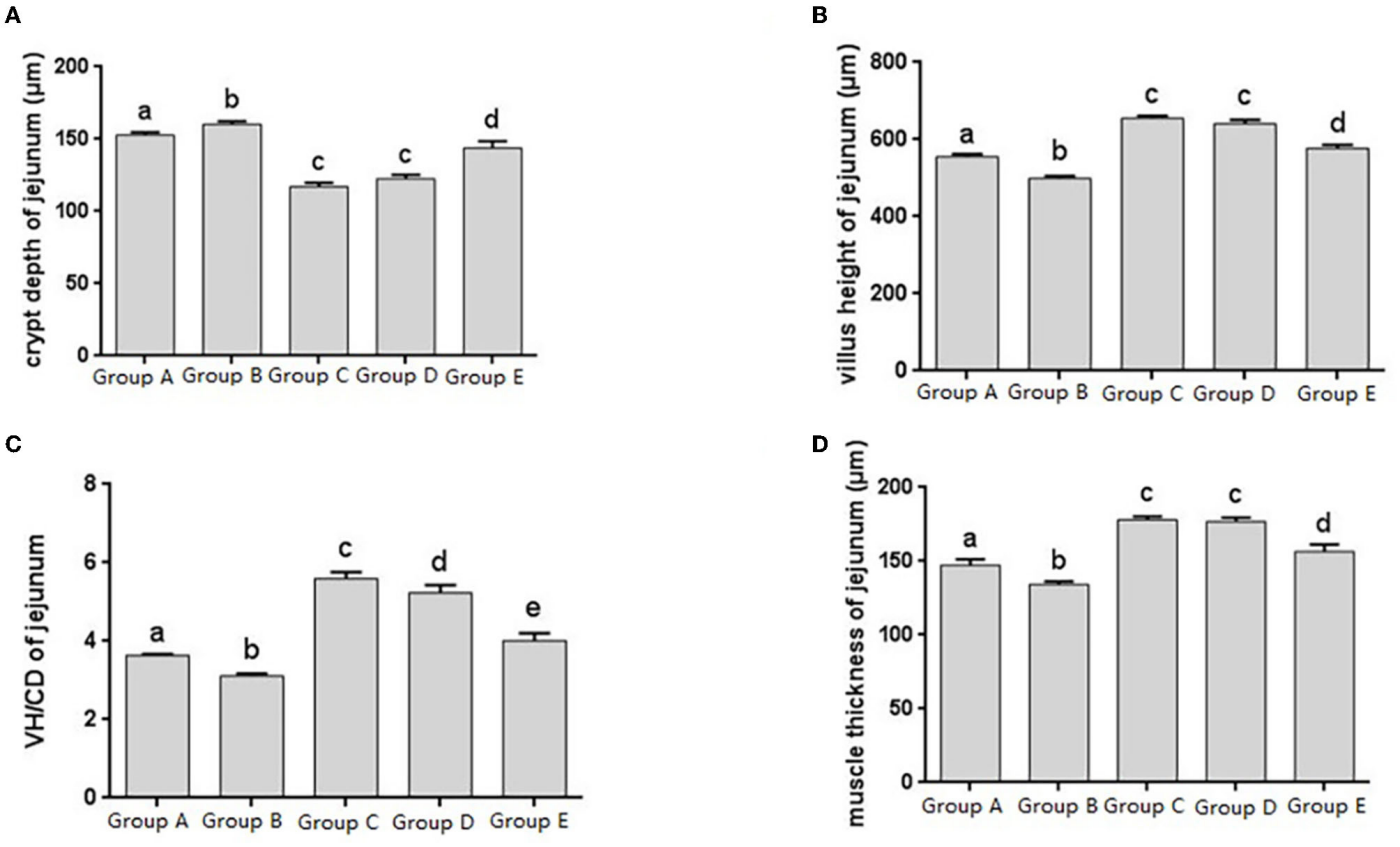
Morphological changes in the jejunm of chicks after different treatments. **(A)** Crypt Depth (CD) of jejunum. **(B)** Villus height (VH) of jejunum. **(C)** VH/CD of jejunum. **(D)** Muscle thickness of jejunum. Group A, control group; Group B, infection group; Group C, JYBR-190 pre-fed group; Group D, JYBR-190 fed group; Group E, antibiotic group. Data are expressed as mean ± standard deviation (SD). Different lowercase letters on the columns indicate significant differences (*P* < 0.05); the same lowercase letters indicate no significant difference (*P* > 0.05).

The jejunal tissue morphology was observed in different treatment groups, and the changes in jejunal villus height, crypt depth, V/C ratio and muscle thickness were measured, and it was found that the change trend was roughly consistent with that of the duodenum (Figures 3A–D). The V/C value of the JYBR-190 pre-fed group was the highest among several (Figure 3C). It indicated that feeding Bifidobacterium lactis JYBR-190 could increase jejunal villus height, V/C value and muscle layer thickness, and reduce intestinal crypt depth. Feeding JYBR-190 before infection could even increase jejunal V/C value.

**Figure 4:**
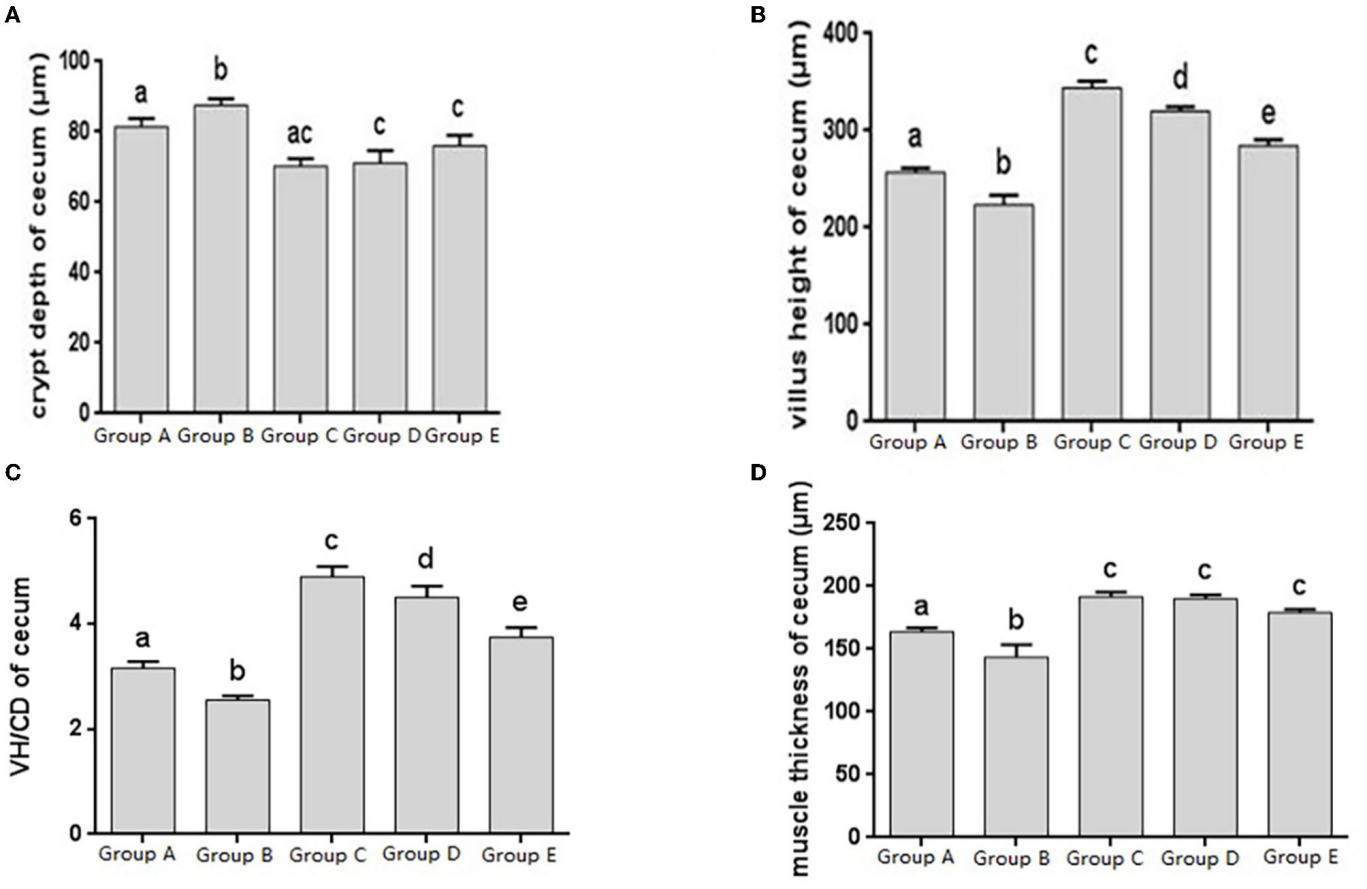
Morphological changes in the cecum of chicks after different treatments. **(A)** Crypt Depth (CD) of the cecum. **(B)** Villus height (VH) of the cecum. **(C)** VH/CD of the cecum. **(D)** Muscle thickness of cecum. Group A, control group; Group B, infection group; Group C, JYBR-190 pre-fed group; Group D, JYBR-190 fed group; Group E, antibiotic group. Data are expressed as mean ± standard deviation (SD). Different lowercase letters on the columns indicate significant differences (*P* < 0.05); the same lowercase letters indicate no significant difference (*P* > 0.05).

The histomorphology of cecum in different treatment groups was observed, and the changes of cecal villus height, crypt depth, V/C ratio and muscle layer thickness were analyzed. Changes in the depth of cecal crypts showed that the depth of intestinal crypts in the Salmonella pullorum infection group was significantly higher than that in the blank control group. Compared with the Salmonella pullorum infection group, the depth of intestinal crypts in the JYBR-190 pre-fed, JYBR-190 fed, and antibiotic groups were reduced to varying degrees, and the difference was significant (Figure 4A). The changes of cecal villus height, V/C value and muscle layer thickness showed that the Salmonella pullorum infection group decreased significantly the intestinal villus height, V/C value and muscle layer thickness compared with the blank control group. Compared with the Salmonella pullorum infection group, the intestinal villus height, V/C value and muscle layer thickness in the JYBR-190 pre-fed, JYBR-190 fed, and antibiotic groups were increased to different degrees, and the difference was significant. Compared with the antibiotic group, the intestinal villi and V/C values of the JYBR-190 pre-fed and fed groups were significantly higher (Figures 4B,C), and the difference in muscle layer thickness was not significant (Figure 4D). Before infection, the intestinal villi and V/C values of the JYBR-190 pre-fed group were the highest among several groups (Figures 4B,C). It indicated that feeding Bifidobacterium lactis JYBR-190 could increase cecal villus height and V/C value and reduce crypt depth, and feeding JYBR-190 before infection could increase cecal villus height and V/C value.

### Changes of PCNA Distribution and Level in Each Intestinal Segment in Different Treatment Groups

**Figure 5:**
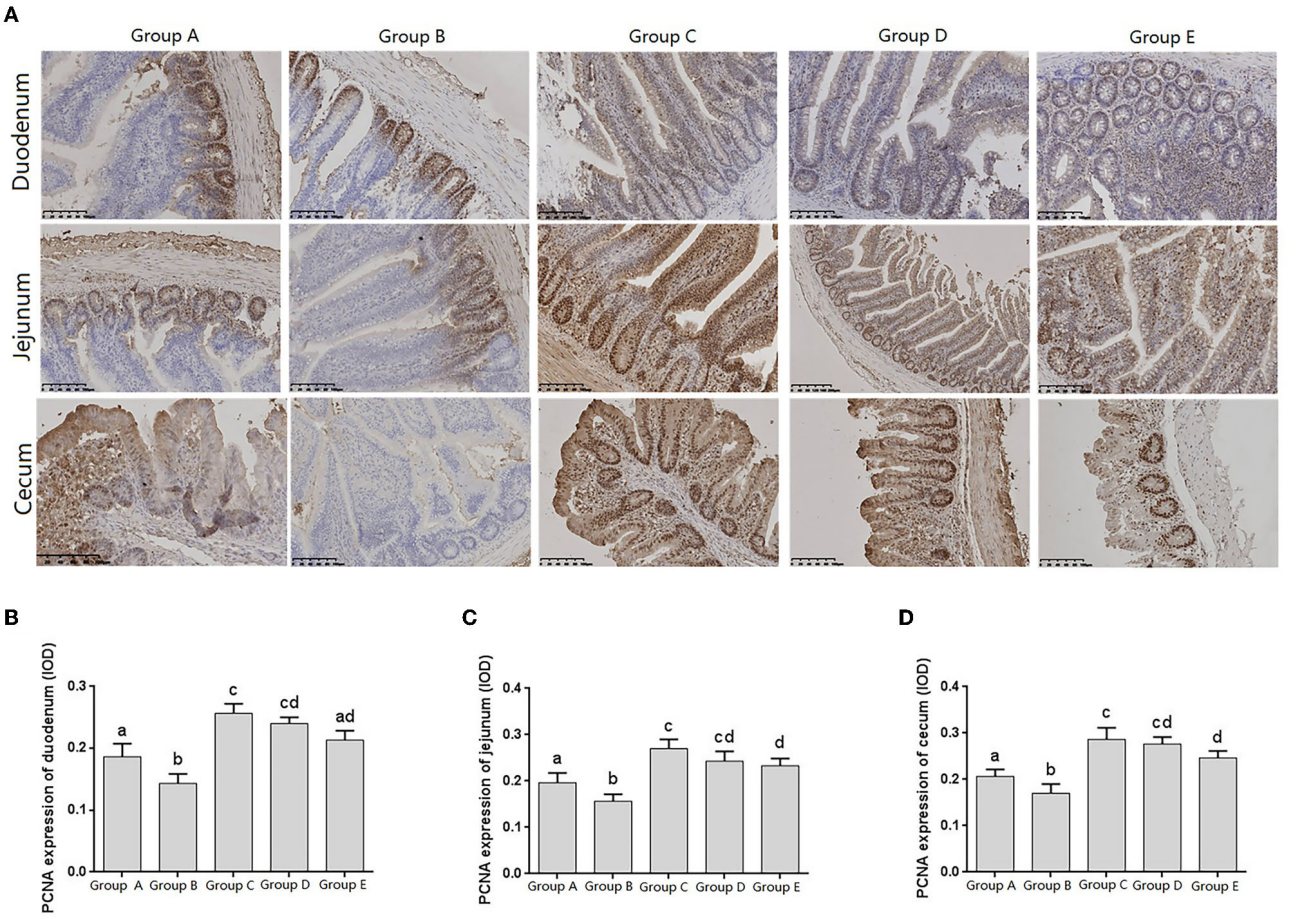
Changes of PCNA distribution in intestine of chicks after different treatments. **(A)** Immunohistochemistry (IHC) staining of the distribution of PCNA in different treatment groups. **(B–D)** Levels of PCNA in different treatment groups. Group A, control group; Group B, infection group; Group C, JYBR-190 pre-fed group; Group D, JYBR-190 fed group; Group E, antibiotic group. Data are expressed as mean ± standard deviation (SD). Different lowercase letters on the columns indicate significant differences (*P* < 0.05); the same lowercase letters indicate no significant difference (*P* > 0.05).

Proliferating cell nuclear antigen (PCNA), as an indicator of cell proliferation status, exists only in normal proliferating cells and tumor cells, and is closely related to cellular deoxyribonucleic acid (DNA) synthesis. The IHC results showed that PCNA was distributed in the nuclei and crypts of the basal epithelial cells of the duodenum, jejunum, and cecum villi (Figure 5A).

The expression levels of PCNA in the duodenum, jejunum and cecum showed that compared with the blank control group, the expression level of PCNA in the infection group was significantly lower. Compared with the infection group, the levels of PCNA in the JYBR-190 pre-fed, JYBR-190 fed, and antibiotic groups were significantly increased. The JYBR-190 pre-fed group was significantly higher than the antibiotic group (Figures 5B–D). It indicated that feeding Bifidobacterium lactis JYBR-190 could increase the expression of PCNA and promote the proliferation and repair of intestinal cells.

### Detection Results of Protein Expression in Intestinal Tissue of Chicks in Different Treatment Groups (ELISA)

#### Changes of Intestinal Cytokine Levels in Different Treatment Groups

**Figure 6:**
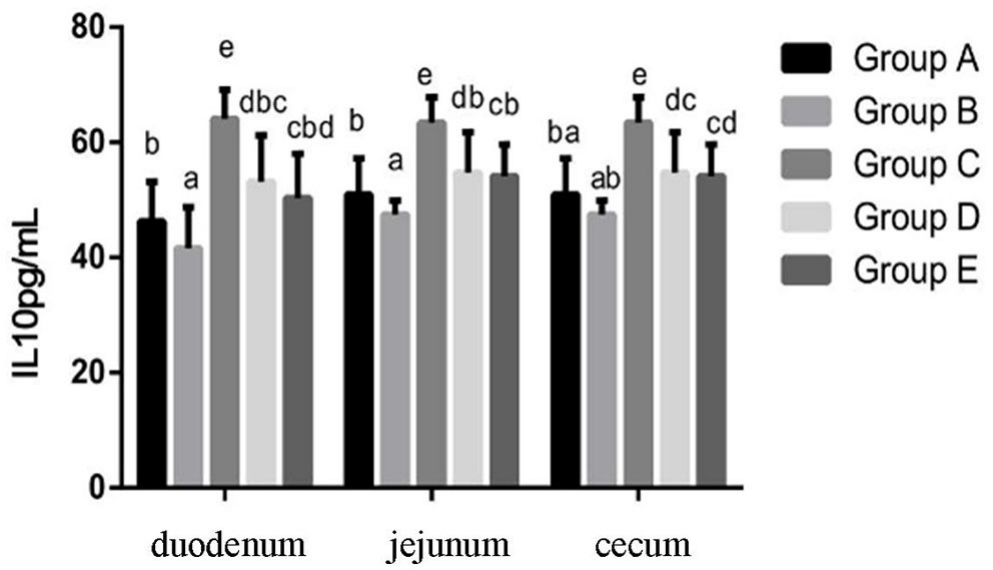
Expression of IL-10 protein in duodenum, jejunum and cecum of different treatment groups. Group A, control group; Group B, infection group; Group C, JYBR-190 pre-fed group; Group D, JYBR-190 fed group; Group E, antibiotic group. Data are expressed as mean ± standard deviation (SD). Different lowercase letters on the columns indicate significant differences (*P* < 0.05); the same lowercase letters indicate no significant difference (*P* > 0.05).

At 14 days of age, the expression levels of IL-10 in the intestinal tissue of chickens with different treatments are shown in Figure 6. The expression levels of IL-10 in the duodenum, jejunum and cecum showed that compared with the blank control group, IL-10 in the infection group was significantly decreased. Compared with the infection group, the level of IL-10 in the JYBR-190 pre-fed, JYBR-190 fed, and antibiotic groups were significantly increased, with the highest level of IL-10 found in the JYBR-190 pre-fed group.

#### Changes of TNF-α Protein Expression Levels in Chicks in Different Treatment Groups

**Figure 7:**
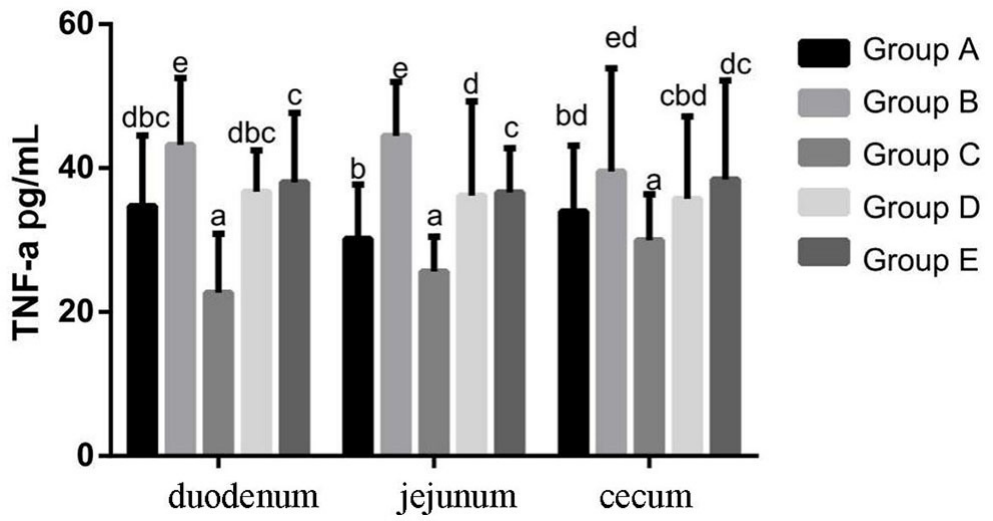
TNF-α protein expression in duodenum, jejunum and cecum of different treatment groups. Group A, control group; Group B, infection group; Group C, JYBR-190 pre-fed group; Group D, JYBR-190 fed group; Group E, antibiotic group. Data are expressed as mean ± standard deviation (SD). Different lowercase letters on the columns indicate significant differences (*P* < 0.05); the same lowercase letters indicate no significant difference (*P* > 0.05).

At 14 days of age, the expression levels of TNF-α protein expression in the intestinal tissue of chickens with different treatments are shown in Figure 7. The expression levels of TNF-α protein expression in the duodenum, jejunum and cecum showed that compared with the blank control group, TNF-α protein expression in the infection group was significantly increased. Compared with the infection group, the level of IL-10 in the JYBR-190 pre-fed, JYBR-190 fed, and antibiotic groups were significantly increased, with the highest level of IL-10 found in the JYBR-190 pre-fed group.

#### Changes of IFN-γ Protein Expression Levels in Chicks in Different Treatment Groups

**Figure 8:**
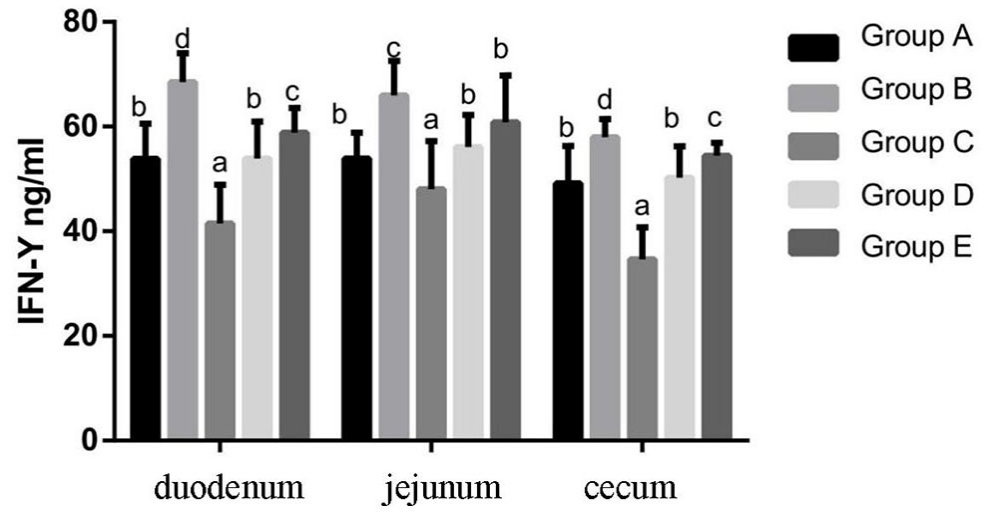
IFN-γ contents of duodenum, jejunum and cecum in different treatment groups. Group A, control group; Group B, infection group; Group C, JYBR-190 pre-fed group; Group D, JYBR-190 fed group; Group E, antibiotic group. Data are expressed as mean ± standard deviation (SD). Different lowercase letters on the columns indicate significant differences (*P* < 0.05); the same lowercase letters indicate no significant difference (*P* > 0.05).

At 14 days of age, the expression levels of IFN-γ protein expression in the intestinal tissue of chickens with different treatments are shown in Figure 8. The expression levels of IFN-γ protein expression in the duodenum, jejunum and cecum showed that compared with the blank control group, IFN-γ protein expression in the infection group was significantly increased. Compared with the infection group, the level of IFN-γ in the JYBR-190 pre-fed, JYBR-190 fed, and antibiotic groups significantly decreased, with the lowest level of IFN-γ found in the JYBR-190 pre-fed group.

#### Changes of SIgA Levels in Chicks in Different Treatment Groups

**Figure 9:**
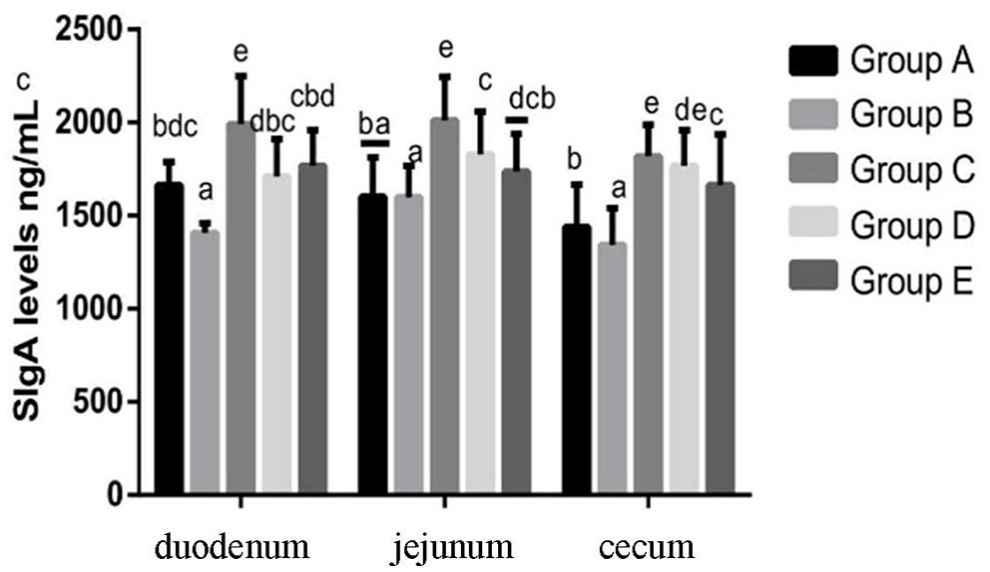
Expression of sIgA Protein in Duodenum, Jejunum and Caecum in different treatment groups. Group A, control group; Group B, infection group; Group C, JYBR-190 pre-fed group; Group D, JYBR-190 fed group; Group E, antibiotic group. Data are expressed as mean ± standard deviation (SD). Different lowercase letters on the columns indicate significant differences (*P* < 0.05); the same lowercase letters indicate no significant difference (*P* > 0.05).

At 14 days of age, the expression levels of sIgA in the intestinal tissue of chickens in different treatment groups are shown in Figure 9. The expression of sIgA in duodenum and jejunum showed that compared with the blank control group, the sIgA in the infection group was significantly decreased. Compared with the infection group, the sIgA level in the JYBR-190 pre-fed, JYBR-190 fed, and antibiotic groups were significantly increased, with the highest sIgA level found in the JYBR-190 pre-fed group. The expression of sIgA in the cecum showed that compared with the blank control group, the sIgA in the infected group was significantly decreased. Compared with the infection group, in the JYBR-190 pre-fed, JYBR-190 fed, and antibiotic groups were significantly increased. Among the three groups, the sIgA level in the JYBR-190 pre-fed and fed groups were significantly higher than that of the antibiotic group. It indicated that feeding Bifidobacterium lactis JYBR-190 increased the expression of sIgA in the duodenum, jejunum and cecum infected with pullorum, and feeding JYBR-190 before infection could promote the expression of sIgA in the duodenum and jejunum.

## Discussion

The duodenum, jejunum and cecum are important components of the intestinal barrier, and the height of the intestinal villi can directly affect the absorption area of the small intestine. The higher the height of the villi, the more mature epithelial absorbing cells, and the better the intestinal digestion and absorption. The depth of crypts reflects the rate of cell formation, and the ratio of villus height to crypt depth (V/C) reflects the strength of intestinal absorption to a certain extent. The higher the ratio, the stronger the intestinal digestion and absorption capacity. Samanya et al. found that Bacillus subtilis can increase the height of ileal villi in adult males and improve feed conversion efficiency (18). In this study, after chicks were infected with Salmonella pullorum, intestinal villus height, villus height ratio (V/C) and muscle thickness in duodenum, jejunum and cecum were significantly decreased, and the depth of intestinal crypts was significantly increased. This indicated that Salmonella pullorum infection caused damage to the intestinal mucosal structure of chicks. However, after treatment with Bifidobacterium lactis JYBR-190 or antibiotics, the villi height, ratio (V/C), and muscle thickness of each intestinal segment were significantly increased, feeding JYBR-190 before infection showed a better repair effect than feeding JYBR-190 and antibiotics after infection. These results indicate that Bifidobacterium lactis JYBR-190 has a protective effect on intestinal mucosal damage and alleviates intestinal inflammation caused by Salmonella pullorum, which is consistent with the function of probiotics such as Bifidobacterium to improve intestinal inflammation.

Intestinal epithelial stem cells are the main repair cells after the intestinal mucosa is damaged, which can maintain the mechanical barrier function of the intestinal mucosa and prevent or reduce the invasion of intestinal bacteria. The expression of PCNA reflects the proliferation status of intestinal epithelial cells and repairability of intestinal mucosal epithelial injury (19). Wu et al. found that in abdominal infection, the role of intestinal mucosal epithelium was obvious, and the expression level of PCNA in the intestine increased, indicating that the proliferation and differentiation activities of intestinal epithelial stem cells were enhanced, thereby promoting the repair of intestinal mucosal damage. However, with infection the further aggravation of the PCNA index indicates that the intestinal epithelial cells are damaged, accompanied by a gradual decline in the proliferative capacity (20). This study found that the expression of PCNA in the duodenum, jejunum and cecum was significantly reduced after Salmonella pullorum infection, indicating that the proliferation and differentiation activities of epithelial stem cells were reduced, causing damage to the intestinal mucosa. The expression of PCNA in the duodenum, jejunum and cecum tissues of chicks fed JYBR-190 increased significantly, indicating that the proliferation and differentiation activities of epithelial stem cells were enhanced, and the repair of intestinal mucosal damage was promoted, indicating that feeding Bifidobacterium lactis JYBR- 190 can increase the expression of PCNA to promote the proliferation and repair of intestinal epithelial cells to maintain the stability of intestinal tissue structure.

Cytokines such as TNF-α, IL-10, and IFN-γ play an important role in the intestinal immune system. The increased secretion of pro-inflammatory factors such as IFN-γ and TNF-α can aggravate intestinal mucosal inflammation and apoptosis of intestinal mucosal epithelial cells. IL-10 is an anti-inflammatory cytokine in the body, which has a blocking effect on the development of immune inflammatory response, and plays a role in maintaining and regulating the intestinal and systemic immune homeostasis. A large number of studies have shown that after chicks are infected with Salmonella, the intestinal mucosa is damaged, resulting in the infiltration of inflammatory cells and the secretion of more inflammatory cytokines, thereby damaging the intestinal mucosa and expanding the chain reaction of intestinal inflammation. In this study, it was found that the expression of TNF-α and IFN-γ was significantly increased, and the expression of IL-10 was significantly decreased after the chicks were infected with Salmonella pullorum, indicating that the infection of Salmonella pullorum resulted in the imbalance of intestinal cell homeostasis and a serious immune disorder. However, after the infected chickens were fed with bifidobacteria, the protein expressions of IFN-γ, TNF-α and other cytokines secreted in the duodenum, jejunum and cecum tissues of chicks were significantly decreased, and the protein expression of IL-10 was significantly increased. In particular, the group fed JYBR-190 before infection was the most significant, indicating that bifidobacteria can inhibit the secretion of pro-inflammatory cytokines and promote the secretion of anti-inflammatory cytokines, thereby maintaining the balance of the intestinal immune system and relieving the intestinal inflammation caused by Salmonella pullorum infection.

sIgA is an important active substance for maintaining gut health, and secretory IgA also regulates the composition and function of gut microbiota, maintaining a mutually beneficial symbiosis between microorganisms and the host (21, 22). According to literature reports, probiotics such as Bifidobacterium lactis, Bifidobacterium bifidum, Lactobacillus and Bifidobacterium lactis can induce the host to secrete sIgA (23–25). After a long period of evolution, pathogenic microorganisms can synthesize an enzyme that hydrolyzes sIgA, which can hydrolyze the sIgA secreted by the intestinal mucosa, which is conducive to the colonization of pathogenic bacteria. In this study, it was found that the expression of sIgA protein in the duodenum, jejunum and cecum of the infected group decreased significantly after the chicks were infected with Salmonella, indicating that the intestinal mucosal immunity of the chicks decreased. However, feeding the chicks with Bifidobacterium lactis JYBR-190 after the expression of sIgA in the duodenum, jejunum and cecum increased significantly. Feeding Bifidobacterium lactis JYBR-190 can increase the expression of sIgA in the intestinal tissue of chicks, and can enhance the intestinal mucosal immunity of chicks to a certain extent.

## Conclusion

Salmonella pullorum infection in chicks damage the intestinal barrier, manifesting as intestinal tissue damage and inflammation, while feeding Bifidobacterium pullorum can significantly reduce the intestinal damage and inflammation in chicks caused by Salmonella pullorum, indicating that Bifidobacterium lactis JYBR−190 has a protective effect on intestinal mucosal damage in chicks infected with Salmonella pullorum.

## Data Availability Statement

The original contributions presented in the study are included in the article/supplementary material, further inquiries can be directed to the corresponding authors.

## Ethics Statement

The animal study was reviewed and approved by Institutional Animal Care and Use Committee of Yunnan Agricultural University.

## Author Contributions

GD, FH, and LY designed the study. LY, YC, QB, XC, and YS performed the study. LY, YC, QB, and RW analyzed the data. LY, GD, and FH wrote the manuscript. All authors read and approved the final manuscript.

## Conflict of Interest

The authors declare that the research was conducted in the absence of any commercial or financial relationships that could be construed as a potential conflict of interest.

## Publisher's Note

All claims expressed in this article are solely those of the authors and do not necessarily represent those of their affiliated organizations, or those of the publisher, the editors and the reviewers. Any product that may be evaluated in this article, or claim that may be made by its manufacturer, is not guaranteed or endorsed by the publisher.
